# Differentiated somatic gene expression is triggered in the dorsal hippocampus and the anterior retrosplenial cortex by hippocampal synaptic plasticity prompted by spatial content learning

**DOI:** 10.1007/s00429-023-02694-z

**Published:** 2023-09-10

**Authors:** Thu-Huong Hoang, Denise Manahan-Vaughan

**Affiliations:** Medical Faculty, Department of Neurophysiology, Ruhr University Bochum, Universitätsstr. 150, MA 4/150, 44780 Bochum, Germany

**Keywords:** Hippocampus, Retrosplenial cortex, Spatial learning, Information encoding, Synaptic plasticity, Immediate early gene

## Abstract

Hippocampal afferent inputs, terminating on proximal and distal subfields of the cornus ammonis (CA), enable the functional discrimination of ‘what’ (item identity) and ‘where’ (spatial location) elements of a spatial representation. This kind of information is supported by structures such as the retrosplenial cortex (RSC). Spatial content learning promotes the expression of hippocampal synaptic plasticity, particularly long-term depression (LTD). In the CA1 region, this is specifically facilitated by the learning of item-place features of a spatial environment. Gene-tagging, by means of time-locked fluorescence in situ hybridization (FISH) to detect nuclear expression of immediate early genes, can reveal neuronal populations that engage in experience-dependent information encoding. In the current study, using FISH, we examined if learning-facilitated LTD results in subfield-specific information encoding in the hippocampus and RSC. Rats engaged in novel exploration of small items during stimulation of Schaffer collateral-CA1 synapses. This resulted in LTD (> 24 h). FISH, to detect nuclear expression of Homer1a, revealed that the distal-CA1 and proximal-CA3 subcompartments were particularly activated by this event. By contrast, all elements of the proximodistal cornus ammonis-axis showed equal nuclear Homer1a expression following LTD induction *solely* by means of afferent stimulation. The RSC exhibited stronger nuclear Homer1a expression in response to learning-facilitated LTD, and to novel item-place experience, compared to LTD induced by sole afferent stimulation in CA1. These results show that both the cornus ammonis and RSC engage in differentiated information encoding of item-place learning that is salient enough, in its own right, to drive the expression of hippocampal LTD. These results also reveal a novel role of the RSC in item-place learning.

## Introduction

The encoding, storage and retrieval of different aspects of memory, has been proposed to occur through concerted interactions of structures such as the hippocampal formation and the retrosplenial cortex (RSC) (Czajkowski et al. 2013; Miller et al. 2014; Mao et al. 2018; Aggleton and Nelson 2015). Within the hippocampus, spatial experience is recorded by means of two persistent forms of synaptic plasticity, namely long-term potentiation (LTP) and long-term depression (LTD) (Manahan-Vaughan 2017, 2018). Whereas LTP is expressed when a rodent is exposed to novel space, or a more general change in allocentric representations (Kemp and Manahan-Vaughan 2004; Straube et al. 2003), LTD is expressed when rodents learn about content details of space (Manahan-Vaughan and Braunewell 1999; Kemp and Manahan-Vaughan 2004, 2008, 2012; Ge et al. 2010; Cui et al. 2013; Dong et al. 2013; Goh and Manahan-Vaughan 2013). Furthermore, a functional differentiation is evident in terms of the kind of spatial content information that facilitates hippocampal LTD: novel learning of, or information updating about, spatial configurations of large features in an environment enables LTD at perforant path dentate gyrus synapses (Kemp and Manahan-Vaughan 2008), and mossy fiber-CA3 synapses (Hagena and Manahan-Vaughan 2011). By contrast, novel learning or information updating about subtle, less ostensible spatial content facilitates LTD at Schaffer collateral-CA1 synapses (Kemp and Manahan-Vaughan 2008), and commissural associational-CA3 synapses (Hagena and Manahan-Vaughan 2011).

Anatomical and neurobiological studies have indicated that the cornus ammonis is functionally segregated in terms of the processing of spatial and identity aspects of item features (Ishizuka et al. 1990; Witter 2007; Henriksen et al. 2010; Ito and Schuman 2012; Hoang et al. 2018). Specifically, distal CA1 and proximal CA3 regions process information about item identity (‘what’ information) and the proximal CA1 and distal CA3 regions process spatial item information (‘where’ information) (Henriksen et al. 2010; Beer et al. 2014; Flasbeck et al. 2018). Gene-tagging, by means of fluorescence in situ hybridization (FISH) to detect nuclear immediate early gene (IEG) expression, that is triggered specifically by these learning events, has confirmed this division of labor in terms of spatial content encoding by hippocampal subregions, and furthermore revealed differences in the engagement of the proximodistal axis of the CA1 region in subtle item-place encoding (Hoang et al. 2018, 2021). Nuclear IEG expression of Arc and Homer1a (Hoang et al. 2018) is specifically triggered in pyramidal cells of the distal-CA1 and proximal-CA3 subfields during learning of spatial locations of small objects that are concealed in holeboard holes, consistent with the processing of ‘what’ information related to item-place experience by these CA subfields (Deshmukh and Knierim 2011; Ito and Schuman 2012; Beer et al. 2013; Nakamura et al. 2013).

The retrosplenial cortex (RSC) supports spatial navigation, spatial memory and spatial cognition (Stacho and Manahan-Vaughan 2022a). Its reciprocal connectivity with the hippocampus forms the function basis for information transfer between both structures. It is subdivided into two subregions, comprising the granular and dysgranular regions (areas 29 and 30) (Sugar et al. 2011). Hippocampal projections to RSC originate in the subiculum, and terminate in layers I, II and III of RSC29 and in layers I and II of RSC30 (Vogt and Miller 1983; Finch et al. 1984; Witter et al. 1990; Naber and Witter 1998; Miyashita and Rockland 2007). Additionally, the dorsal CA1 also projects to layer II, III and IV of RSC29, but not RSC30 (Meibach and Siegel 1977; Naber and Witter 1998; van Groen and Wyss 2003; Miyashita and Rockland 2007). In turn, direct RSC projections to the hippocampal formation originate in layer V of the RSC 29, and terminate only in the subiculum (van Groen and Wyss 1990a, 2003; Shibata 1994). Furthermore, projections from areas 29 and 30 of the RSC, mostly originating in layers II and V, extend to all subdivisions of the parahippocampal areas, including the postrhinal cortex, perirhinal cortex and entorhinal cortex (EC), which, in turn, send projections to the hippocampus (Deacon et al. 1983; Guldin and Markowitsch 1983; Wyss and van Groen 1992; Shibata 1994; Burwell and Amaral 1998; Jones and Witter 2007). The postrhinal and perirhinal cortices project directly to both areas 29 and 30 of the RSC (Agster and Burwell 2009). Furthermore, back projections from neurons in layer V of the EC terminate mostly in layers I, II and IV of RSC29 and in layers I and II of RSC30 (Fröhlich and Ott 1980; Insausti et al. 1997; Agster and Burwell 2009).

In addition to its projections to the hippocampus, the RSC also communicates with other structures, such as the visual cortex (V2 and V4) and the parietal cortex that are critical for the encoding of visual and sensory information (Olsen et al. 2017; Clark et al. 2018; Mao et al. 2020), that, in turn, is used by the hippocampus to record spatial experience (Kemp and Manahan-Vaughan 2004; Tsanov and Manahan-Vaughan 2009; André and Manahan-Vaughan 2013; Dietz and Manahan-Vaughan 2017). Areas 29 and 30 of the RSC may contribute differently to spatial learning and navigation-based information acquisition, however. Whereas the contribution of RSC29 to memory performance depends on both allocentric and idiothetic cues (Pothuizen et al. 2009), RSC30 may be particularly involved in the processing of allocentric information and play a role in cross-modal object recognition (Vann and Aggleton 2005; Hindley et al. 2014a, b). In line with this, functional inactivation of the RSC leads to impaired performance in rodents in standard spatial memory tasks, e.g. the use of distal (allocentric) visual cues, the use of directional information, and the use of idiothetic information for path integration (Cooper and Mizumori 1999, 2001; Alexander and Nitz 2017; van Wijngaarden et al. 2020). In addition, area 29 of the anterior RSC has been implicated in the processing of spatial (“where”) and nonspatial (“what”) memory (Landeta et al. 2020). So far, most behavioral studies in rodents have used spatial visual directional and landmark cues to examine the involvement of the RSC in spatial navigation or spatial recognition (Stacho and Manahan-Vaughan 2022a). It is however as yet unclear, whether the RSC is also required for the encoding and processing of information that is specifically encoded by the hippocampus, such as item-place spatial content (Hoang et al. 2018, 2021). In this study we therefore explored to what extent the cornus ammonis and RSC contribute to the encoding of item-place experience, that is known to facilitate the expression of hippocampal LTD.

## Materials and methods

### Electrophysiology

The study was conducted in accordance with the European Communities Council Directive of September 22nd, 2010 (2010/63/EU) for care of laboratory animals and after approval of the state ethics committee (Landesamt für Arbeitsschutz, Naturschutz, Umweltschutz und Verbraucherschutz, North Rhein-Westfalia). All efforts were made to minimize the number of rats used for this study. The animals were housed in groups before, and in single cages after, surgery in a temperature and humidity-controlled vivarium (Scantainer Ventilated Cabinets, Scanbur A/S, Denmark) with a constant 12-h light–dark cycle (lights on from 7 a.m. to 7 p.m.) and controlled temperature (22 ± 2 °C) and humidity (55 ± 5%). Food and water were available ad libitum throughout all experiments.

**Fig. 1:**
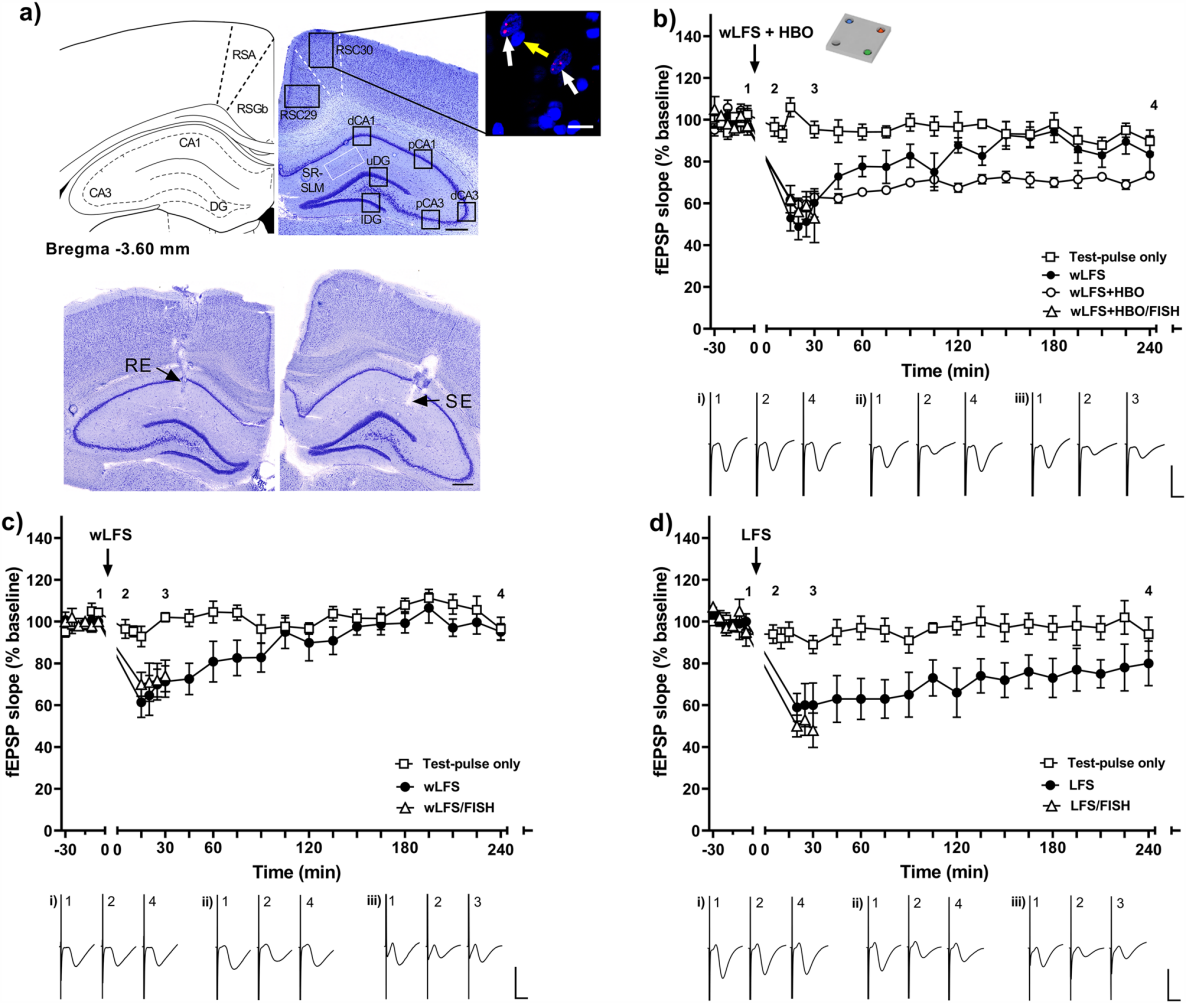
Location of regions of interest and in vivo electrophysiological recordings. **a** Top: Schema of analyzed regions of interest in a coronal section of rat brain. Top Left: The schema shows a section through the rat brain at the level of the dorsal hippocampus (ca. 3.60 mm posterior to Bregma) that highlights the cornu ammonis (CA), dentate gyrus (DG) and the agranular (RSA) and granular (RSGb) parts of the retrosplenial cortex (RSC). Top right: Nissl-stained image of a coronal section representing the location of z-stacks (black square frames) obtained for the RSC areas 29 and 30, distal CA1 (dCA1), proximal CA1 (pCA1), distal CA3 (dCA3), proximal CA3 (pCA3), upper blade (uDG) and lower blade (lDG) of the Dentate Gyrus (DG) and at the border of stratum radiatum (SR) and Stratum lacunosum-moleculare (SLM) (white square frame). Scale bar 500 µm. Top right inset: Example of nuclear Homer1a mRNA signals obtained in nuclei of neurons within RSC30. Only neurons that contained Homer1a mRNA (red dots) inside the nuclei (blue-stained by DAPI) were counted as positive (indicated by white arrows). A glial cell is indicated by the yellow arrow. Image magnification was obtained at final magnification 20 ×. Scale bar: 20 µm. Bottom: Representative Nissl-stained images showing positions of implanted electrodes in the dorsal hippocampus. The stimulating electrode (SE) was implanted in the Schaffer collaterals of the right hemisphere and the recording electrode (RE) was placed in the stratum radiatum of the contralateral hemisphere. Scale bar: 500 µm. **b** Test-pulse stimulation evoked potentials that were stable for the duration of recordings. Weak low frequency stimulation (wLFS, 1 Hz, 600 pulses) Schaffer collaterals triggered STD in the stratum radiatum that lasted for < 90 min. Exploration of novel objects present in holeboard holes (HBO) in conjunction with LFS resulted in LTD that lasted for > 4 h. For FISH, in another cohort the wLFS + HBO experiment was terminated after verification that synaptic depression had been triggered. **c** In a further animal cohort, after verification of stable baseline responses in a test-pulse experiment and subsequent verification that wLFS induces STD (< 90 min), wLFS was repeated 7–10 days later and terminated after verification that synaptic depression had been triggered, so that FISH could be conducted. **d** After verification of stable synaptic transmission in a test-pulse experiment, LFS (1 Hz, 900 pulses) was applied to the Schaffer collaterals to induce LTD in the stratum radiatum. After a period of 7–10 days and after verification that evoked responses had return to pre-LFS levels, the LFS experiment was repeated so that FISH could be conducted. In **b**–**d**, analog traces were recorded from (i) an animal that received test-pulse (test-pulse only), (ii) from the same animal when afferent stimulation was applied on the first day (wLFS or LFS) and (iii) after 3–7 days (wLFS/FISH or LFS/FISH or wLFS + HBO). Examples show fEPSPs recorded at the time points signified by numbers in the graphs. Horizontal scale bar: 10 ms, vertical scale bar: 3 mV

Under sodium pentobarbital anesthesia (52 mg/kg animal weight, intraperitoneally), 7–8 week old male Wistar rats underwent chronic implantation of stimulating and recording electrodes into the Schaffer collaterals and Stratum radiatum of the dorsal hippocampal CA1 region, respectively, as described previously (Kemp and Manahan-Vaughan 2012). The recording electrode was placed in the Stratum radiatium of the CA1 region of the left dorsal hippocampus (2.8 mm posterior, 1.8 mm lateral to bregma, Fig. 1a), whereas the stimulating electrode was placed in Schaffer collateral/commissural fibers of the right hippocampus (3.1 mm posterior, 3.1 mm lateral to bregma, Fig. 1a). This was done to avoid unnecessary damage to the hippocampus, especially because of subsequent FISH analysis. We have previously shown that contralateral stimulation of the Schaffer collateral/commissural fibers triggers LTD in the ipsilateral CA1 region (Kemp and Manahan-Vaughan 2012). Animals recovered for 7–10 days after surgery before the commencement of experiments. In all cases, the animals were placed in the recording chambers 24 h before the experiment began, to allow acclimatization to the chamber.

During experiments, animals could freely move in the walled recording chambers that were 40 × 40 × 40 cm in size, open at the top, and were made of gray washable acrylic panels (perspex). The chamber interior was accessible via a removable translucent perspex front wall. During recordings, the implanted electrodes were connected to the recording and stimulation equipment by a flexible cable and a commutator (Fine Science Tools, USA). Recordings were obtained in CA1 stratum radiatum by stimulating the Schaffer collateral/commissural fibers. The maximum slope of the field excitatory postsynaptic potentials (fEPSP) was calculated by means of an input–output (I/O) relationship that was conducted on the morning of each experiment (100- max. 900 μA, in steps of 100 μA). The intensity of the stimulation was set at 40% of the maximal fEPSP slope obtained. Test-pulses were applied at 0.025 Hz. Each time-point was determined from the average of five consecutive test-pulses. The first six time-points of the experiment were recorded every 5 min and served as a baseline reference for any further changes in synaptic strength during subsequent recordings (that were calculated as a percentage of this baseline).

At least 2 weeks before starting the main experiments, assessments of the stability of basal synaptic transmission were assessed over a 4 h period (Fig. 1b–d). Only those animals that showed an average baseline response that remained stable, during this period were used in experiments. These animals were then tested for their ability to express short-term depression (STD) that lasts for < 90 min, or long-term depression (LTD) that lasts for > 4 h. For this, after 30 min of baseline recordings, weak low frequency stimulation (wLFS), consisting of 1 Hz, 600 pulses, was applied to induce STD (Fig. 1b, c) or more prolonged low frequency stimulation of 1 Hz, 900 pulses (LFS) was applied to induce LTD (Fig. 1d). During wLFS, or LFS, the stimulation intensity was increased to 70% of the maximal fEPSP, as determined by the I/O relationship of the individual animal (Kemp and Manahan-Vaughan 2008). Beginning 5 min after the conclusion of wLFS or LFS, fEPSP recordings were obtained at 5 min intervals for a period of 15 min. Immediately afterwards, the recording interval was increased to 15 min intervals. Recordings were concluded 4 h after wLFS or LFS had been applied. Only those animals that expressed synaptic depression proceeded to the spatial content experiments that were conducted 7–10 days after LFS experiments, and after confirmation (prior to beginning each experiment) that the I/O relationship had returned to pre-LFS levels. In two other animal cohorts, wLFS, or LFS, were applied to induce STD or LTD, respectively, and then the brains were removed and shock frozen for subsequent fluorescence in situ hybridization (FISH).

### Spatial content experiments

All animals experienced handling and habituation by the experimenter for several days prior to the commencement of the experiments. The experiments were conducted in the same recording chambers described above. Basal synaptic transmission was recorded for 30 min and then a holeboard (39.8 cm width × 39.8 cm length, gray perspex) was inserted to the chamber (see inset to Fig. 1b). The holeboard contained four holes that were equidistant from one another and were accessible from all areas of the holeboard. In three of the four holeboard holes (5.5 cm in diameter and 5 cm deep) a novel object was placed, as described previously (Kemp and Manahan-Vaughan 2004, 2008). The objects did not extend above the surface of the holeboard floor. Rather, the animals had to insert their noses into the holes to discover the objects. At the time-point of holeboard insertion, wLFS was applied. The holeboard remained in the chamber for the duration of wLFS and was then removed. Five minutes after the conclusion of wLFS, potentials were evoked by test-pulse stimulation and recorded as described above.

In an additional cohort, animals explored the holeboard, containing small objects in the holeboard holes, without receiving any afferent stimulation. These animals did not undergo electrode implantation, so that we could assess the effect of learning (in the absence of any other manipulation) on IEG expression. Animals were habituated to the recording chambers (as described above) for 1 h/day for 3 consecutive days prior to acquisition. On the day of the experiment, they resided in the test chamber for 1 h before the experiment was conducted. After the habituation, a holeboard containing small objects (as described above) was inserted to the chamber for a 10 min exploration event and was subsequently removed from the chamber.

Object exploration was video-monitored, and the experiment was discontinued, or the data discarded, if the rat spent less than 5 min exploring the objects.

### Fluorescence in situ hybridization *(FISH)*

Homer1a plays an important role in the neuroplastic mechanisms critical to memory consolidation (Clifton et al. 2019). In rodents, its nuclear expression peaks 30–40 min after an experience-dependent triggering event (Bottai et al. 2002; Vazdarjanova et al. 2002). Thus, due to its narrow, activity-dependent and specific expression, nuclear Homer1a mRNA has been used as a biomarker to identify the neuronal populations that specifically engage in experience encoding (Vazdarjanova et al. 2002; Hoang et al 2018, 2021). We conducted FISH to detect nuclear Homer1a expression that was triggered by wLFS, LFS, or wLFS coupled with item-place learning in the abovementioned holeboard. Brains were removed 40 min after each event commenced and were rapidly shock frozen.

The following groups of animals (n = 6 each) were differentiated:

1. Electrodes present, no test-pulse stimulation, no learning event (control). Animals remained in the recording chamber for an equivalent amount of time as the spatial content exploration group.
2. Exploration of items concealed in holeboard holes in conjunction with wLFS (wLFS + HBO) (Fig. 1b).
3. wLFS only to induce STD (Fig. 1c).
4. LFS only to induce LTD (Fig. 1d).
5. Exploration of items placed in holeboard holes without electrophysiological stimulation (HBO).
6. No electrodes present, no exploration event (control, for HBO).

Brains were rapidly removed, quick-frozen in isopentane (− 80 °C) and stored at − 80 °C until slicing into sections using a cryostat (Leica CM 3050S, Leica Biosystem GmbH, Wetzlar, Germany). For this, 20 µm thick coronal sections (3 slices per glass slide) containing the hippocampus and the RSC (from ca. 2.8 to 4.8 mm posterior to Bregma) were collected, mounted directly on object slides (SuperFrost®Plus, Gerhard Menzel GmbH, Braunschweig, Germany) and stored afterwards at − 80 °C for further processing. Nissl sections were additionally collected (every third slide from the serial sections), in order to verify the anatomical regions, as well as the quality of the sections.

Digoxigenin-labeled probes were created using the Ambion MaxiScript Kit (Invitrogen, ThermoFisher Scientific, Waltham, Massachusetts, USA). Homer1a cDNA plasmid was prepared commercially (Genscript Biotech, Piscataway Township, New Jersey, USA) using a ~ 1.2 kb Homer1a transcript (Brakeman et al. 1997). The cRNA probes were prepared from the linearized cDNA using a transcription kit (Invitrogen Ambion MaxiScript Kit, ThermoFisher Scientific Waltham, USA) and a premixed RNA labeling nucleotide mix containing the Digoxigenin-11-UTP (Roche Diagnostics, Basel, Switzerland). Generated RNA probes were purified on Mini Quick Spin RNA columns (Roche Diagnostics, Basel, Switzerland). Their yield and integrity were verified using gel electrophoresis and the concentration was measured by using a QuantiFlour® RNA system (Promega, Madison, USA).

For FISH, we chose one glass slide per animal (including three consecutive brain sections each) that was then left at room temperature (RT) until the slices were defrosted. From each animal, the glass slide containing the dorsal hippocampal, as well as RSC sections at ca. 3.60 mm posterior to Bregma was chosen, by comparing the sections to the standardized appearance of brain sections in a rat atlas (Paxinos and Watson 2014). We then applied the FISH protocol, as described previously (Hoang et al. 2021; Strauch et al. 2022) for identification of nuclear Homer1a mRNA expression. For this, brain sections were fixed in ice-cold 4% paraformaldehyde in fresh and filtered phosphate buffered saline (PBS), washed in twofold concentrate saline-sodium citrate buffer (2xSSC), then incubated in acetic anhydride solution. Slides were quickly washed in 2xSSC and incubated with prehybridization buffer (Sigma-Aldrich, St. Louis, USA) at RT. The digoxigenin-labeled Homer1a RNA probe was diluted with a concentration of 1 ng/1 µl in hybridization buffer (Sigma-Aldrich, St. Louis, USA), heated at 90 °C for 5 min, then applied on the brain sections, and hybridized for approximately 17 h in a humid chamber at 56 °C. To confirm the specificity of the hybridized signal, we additionally conducted a negative control FISH test whereby the digoxigenin labeled Homer1a RNA was not added to the brain sections (data not shown). Following the hybridization, the treatment with RNase A and stringent washing steps were conducted, and slides were then incubated with H_2_O_2_ solution in order to block the endogenous peroxidase. Homer1a mRNA signal was detected by anti-digoxigenin-peroxidase Fab fragment from sheep (Roche Holding AG, Basel, Switzerland), enhanced by using biotinylated tyramine, and visualized using Streptavidin Cy5 (Dianova, Hamburg, Germany). Afterwards, slides were quickly rinsed in distilled water, dipped in 70% ethanol, and stained using 1% Sudan black B (Merck KGaA, Darmstadt, Germany) in 70% ethanol (Oliveira 2010). Slides were then rinsed in distilled water, air dried overnight and mounted in antifading mounting medium (immunoSelect®, Dianova, Hamburg, Germany) containing 4′-6-diamidino-2-phenylindole (DAPI).

### Data analysis

To determine the relative expression of Homer1a mRNA in the regions of interest (ROIs), images were acquired in the right hemisphere of the brain slices, where the stimulation electrode was placed. The contralateral electrode was used to verify that STD, or LTD, had occurred, or that basal synaptic transmission was stable in control experiments. To analyze Homer1a mRNA expression within the nuclei of the CA1 and CA3 pyramidal cells and dentate gyrus (DG) granule cells, z-stacks were obtained at a 63 × magnification using an Apotome fluorescence microscope (Zeiss, Oberkochen, Germany). The regions of interest (ROIs) examined comprised the distal and proximal CA1 regions (dCA1 and pCA1), the distal and proximal CA3 regions (dCA3 and pCA3) and the combined lower and upper layers of the DG (Fig. 1a). For the anterior RSC, z-stacks were captured in the granular (RSC29 or RSG) and dysgranular (RSC30 or RSA) areas at a final magnification of 20 × (Fig. 1a). At the border of stratum radiatum and stratum lacunosum-moleculare, z-stacks were obtained at a final magnification of 20 × (Fig. 1a). Using ImageJ software (Schindelin et al. 2012), the complete nuclei were manually marked. The nuclei that contained the Homer1a mRNA signal (Fig. 1a, microphotograph, white arrows) were counted as positive. The percentages of the Homer1a mRNA positive nuclei were calculated relative to the total number of DAPI-labeled nuclei, for one ROI per section. Glial cells (Fig. 1a, yellow arrow) were excluded from analysis. They were identified on the basis of their more intense nuclear staining with DAPI and by the fact that their nuclei are typically < 8 µm in size. One hemisphere of each of three consecutive brain slices from each animal was analyzed and the average percentages of positive Homer1a mRNA nuclei of three slices were calculated. Then for each region, the average Homer1a expression of each group of animals are presented as mean percentage ± SEM. The analysis was conducted in an experimenter-blind manner.

### Statistical analysis

All values were verified for normal distribution using the Kolmogorov–Smirnov test. For statistical analysis Statistica software (STATISTICA Version 14.0.0.15 RRID:SCR_014213) was used. For electrophysiological experiments, the interaction effect between afferent stimulus, with or without a spatial learning task, and baseline values (test-pulse only) was analyzed by means of repeated measures analysis of variance (rmANOVA) followed by a Fisher LSD post-hoc test. For FISH, a multifactorial ANOVA (mANOVA) or one-way ANOVA was conducted to examine the significant effect under different experimental conditions on the expression of Homer1a mRNA. Then, subsequent post-hoc tests (Tukey HSD and Fisher LSD) were conducted to assess further subregion-specific effects in control animals, and in animals under test conditions. The significance level was set at *p* < 0.05.

## Results

### Hippocampal afferent stimulation during novel item-place learning facilitates the expression of LTD

In freely behaving animals, test-pulse stimulation evoked fEPSPs that remained stable for the duration of the experiment (Fig. 1b–d). Weak low frequency stimulation (wLFS, 1 Hz, 600 pulses) resulted in short-term depression (STD) that lasted for < 90 min compared to test-pulse stimulated controls (Fig. 1b, rmANOVA, F_(1,10)_ = 11.1437, *p* = 0.0075, n = 6 each). Seven-to-ten days after this initial wLFS experiment, the same animals received wLFS in conjunction with novel exposure to items in holeboard holes (HBO) followed by brain removal for FISH analysis of nuclear Homer1a expression. Here significant synaptic depression was also induced (Fig. 1b, test-pulse only vs. wLFS + HBO/FISH, rmANOVA F_(1,10)_ = 38.9658, *p* = 0.0001, n = 6). In another cohort of animals we also confirmed that wLFS of Schaffer collateral (SC)-CA1 synapses, in conjunction with novel exposure to HBO results in LTD that lasts for > 4 h (Fig. 1b wLFS + HBO vs. wLFS, rmANOVA F_(1,10)_ = 8.2431, *p* = 0.0166), as reported previously (Manahan-Vaughan and Braunewell 1999; Kemp and Manahan-Vaughan 2004, 2008).

In the next cohort of animals, we applied wLFS (1 Hz, 600 pulses) in the absence of a behavioral event, to induce STD (Fig. 1c) compared to test-pulse stimulated controls (rmANOVA F_(1,10)_ = 7.513, *p* = 0.0208, n = 6 each). Following this confirmation that the animals express STD (< 90 min), we repeated the experiment 7–10 days later and once more successfully induced STD (Fig. 1c, rmANOVA F_(1,10)_ = 10.2865, *p* = 0.0094, n = 6), but this time brain removal, for FISH analysis of nuclear Homer1a expression, was conducted.

In a third cohort of animals, we initially confirmed that LTD was induced by applying LFS (1 Hz 900 pulses) to SC-CA1 synapses (Fig. 1d). Here, LTD was expressed that lasted for over 4 h compared to test-pulse stimulated controls (rmANOVA F_(1,10)_ = 9.6041, *p* = 0.011269, n = 6). Seven to 10 days later the experiment was repeated followed by brain removal, for FISH analysis of nuclear Homer1a expression. Here too, significant synaptic depression was evident compared to controls (rmANOVA: F_(1,8)_ = 28.9945, *p* = 0.000658, n = 6 for test-pulse only and n = 4 for LFS/FISH).

### Exposure to novel item-place during weak low frequency stimulation leads to a differentiated increase in Homer1a mRNA expression in the CA1 and CA3, but not dentate gyrus regions

**Fig. 2:**
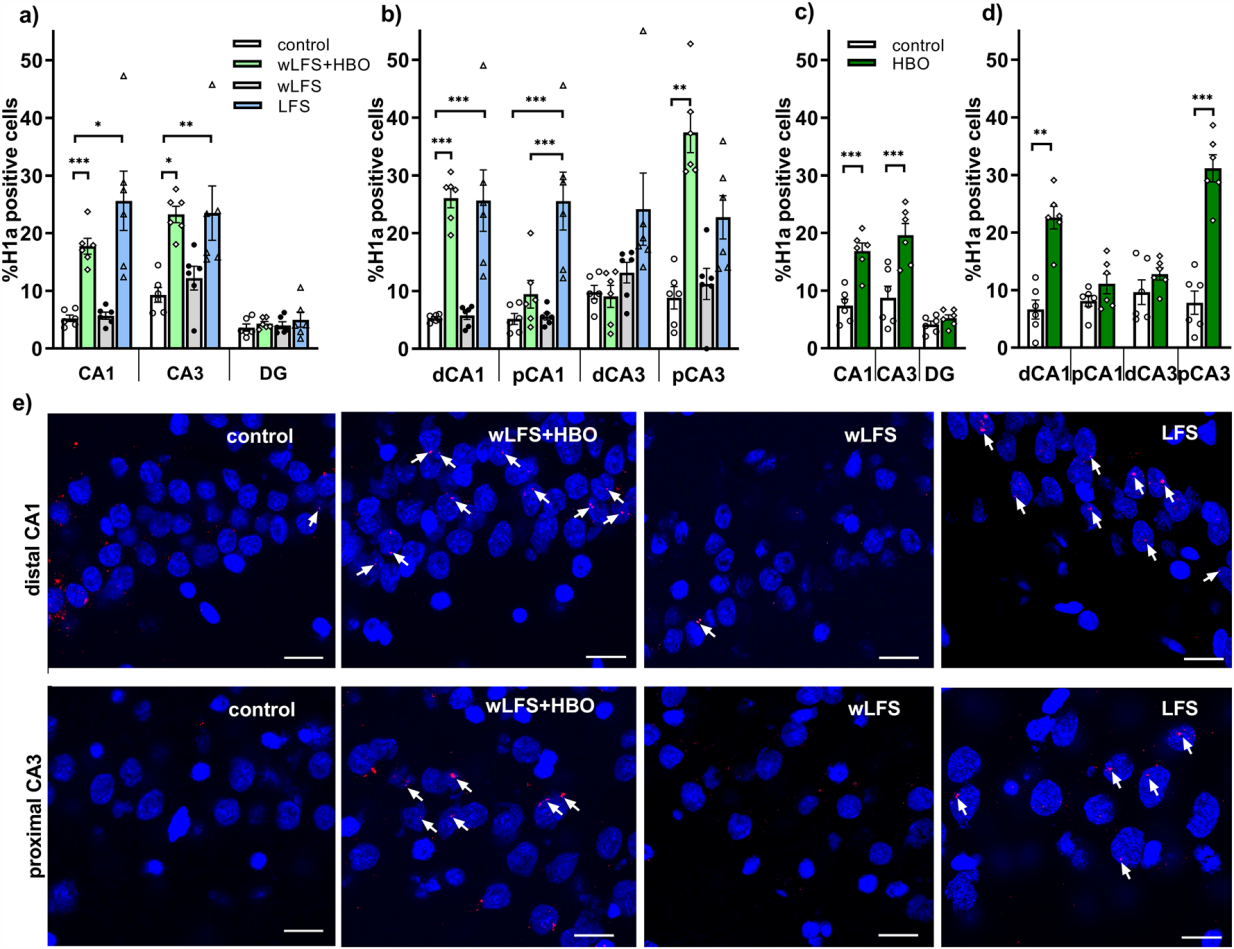
Significant elevations of nuclear Homer1a mRNA expression occur in the cornus ammonis subregions following the induction of long-term depression. **a**, **b** Bar charts show the relative percentage of Homer1a mRNA expression (mean ± SEM) in the hippocampal subregions after wLFS, wLFS + HBO and LFS, compared to control animals (control). **a** Following wLFS, no significant changes in Homer1a mRNA expression were observed in the CA1, CA3 subregions and the DG. By contrast, significant elevations of nuclear Homer1a expression occurred in the CA1 regions and CA3 regions was detected after LFS compared to control animals. Exploration of a holeboard, containing novel items placed inside the holeboard holes (HBO) during wLFS, significantly increased Homer1a mRNA expression in distal CA1 and proximal CA3 subfields compared to controls. No expression difference was observed in the DG, compared to control animals. **c**, **d** Relative percentages of Homer1a mRNA expression (mean ± SEM) in the hippocampal subregions triggered by the acquisition of novel item-place information (HBO) in the absence of electrophysiological manipulations, compared to controls. Exposure to HBO significantly elevated Homer1a mRNA expression in the distal CA1 and proximal CA3 regions whereas no significant differences was detected in the DG, compared to control animals. *p < 0.05, **p < 0.01, ***p < 0.001. **e** Photomicrographs show examples of increased Homer1a expression in the distal CA1 and proximal CA3 in response to novel exploration of spatial microscale cues (wLFS + HBO) and to LFS (1 Hz, 900 pulses) compared to the responses elicited by wLFS (1 Hz, 600 pulses), or responses detected in controls. FISH images were obtained using a 63 × objective. Nuclei were stained with DAPI (blue). Homer1a signals (red dots) are indicated by white arrows. Scale bar: 20 μm

FISH, to detect nuclear expression of Homer1a in the cornus ammonis,  revealed a significant difference in expression following exposure of the animals to wLFS in conjunction with novel HBO exposure (Fig. 2a, b, e), compared to responses elicited in the control condition, by wLFS, or LFS, all applied in the absence of HBO (control vs. wLFS + HBO vs. wLFS vs. LFS, mANOVA F_(3,60)_ = 19.9951, *p* = 0.0000, see also Table 1a–c). Here, subfield specific expression was evident, whereby the distal CA1 and proximal CA3 subfields were strongly activated by the wLFS + HBO event compared to controls (Fisher LSD post-hoc test *p* = 0.0001 for dCA1 and pCA3, n = 6 each). By contrast, no significant change in nuclear Homer1a expression was detected in the proximal CA1 and distal CA3 subfields (Fig. 2b; Table 1a, b, n = 6 each). Further post-hoc comparisons revealed that the Homer1a mRNA expression responses of the distal CA1 and the proximal CA3 regions were significantly greater than in the proximal CA1 and distal CA3 regions (Table 1c). By contrast, IEG expression was unchanged in the DG of wLFS + HBO animals compared to controls (Fig. 2a; Table 1, n = 6). Given that the Schaffer collaterals do not project to the DG (Amaral and Witter 1989), and that the DG does not encode discrete spatial (item-place) content (Kemp and Manahan-Vaughan 2008; Hoang et al. 2018), these latter results were unsurprising. Thus, wLFS during novel item-place learning results in a differentiated increase in Homer1a expression that is specific to the distal CA1 and proximal CA3 subfields of the hippocampus.

**Table 1:** Summary of statistical analysis of nuclear Homer1a expression in the hippocampus during afferent stimulation

	wLFS versus control	wLFS + HBO versus control	LFS versus control	wLFS + HBO versus wLFS	LFS versus wLFS
*(a)*					
**CA1**	*p* = 1.0000	***p* = 0.0119**	***p* = 0.0001**	***p* = 0.0182**	***p* = 0.0001**
**CA3**	*p* = 0.9989	***p* = 0.0030**	***p* = 0.0024**	***p* = 0.04**	***p* = 0.033**
**DG**	*p* = 1.0000	*p* = 1.0000	*p* = 0.9999		
*(b)*					
**dCA1**	*p* = 0.8953	***p* = 0.0000**	***p* = 0.0000**	***p* = 0.0000**	***p* = 0.0000**
**pCA1**	*p* = 0.9048	*p* = 0.2365	***p* = 0.0000**	*p* = 0.2868	***p* = 0.0000**
**dCA3**	*p* = 0.3480	*p* = 0.8384	***p* = 0.0001**	*p* = 0.2538	***p* = 0.0025**
**pCA3**	*p* = 0.5070	***p* = 0.0000**	***p* = 0.0001**	***p* = 0.0000**	***p* = 0.0017**

	wLFS	wLFS + HBO	LFS
*(c)*			
**dCA1 versus pCA1**	*p* = 0.9593	***p* = 0.0000**	*p* = 0.9863
**dCA3 versus pCA3**	*p* = 0.5866	***p* = 0.0000**	*p* = 0.6781

Statistical outcome of (a) between-group comparisons for each hippocampal subfield using Tukey HSD post hoc test; (b) between-group comparisons for each hippocampal subcompartment using Fisher LSD post hoc test; (c) between-subcompartment comparisons using Fisher LSD post hoc test

Significant effects (*p* < 0.05) are highlighted in bold font. All groups include data from n = 6 animals

*dCA1* distal CA1, *dCA3* distal CA3, *pCA1* proximal CA1, *pCA3* proximal CA3, *DG* dentate gyrus, *HBO* holeboard including novel objects, *LFS* low frequency stimulation, *wLFS* weak low frequency stimulation

To establish whether wLFS during item-place learning is essential for LTD induction that leads, in turn, to the detected increase in Homer1a expression in the CA1 region, we repeated the HBO experiment in the absence of any electrophysiological manipulations. The corresponding control cohort of animals was placed in the recording chamber under identical experimental conditions, but did not experience an item-place event. In animals that were exposed to novel HBO only, we observed a significant elevation of Homer1a mRNA expression in the CA1 and CA3 regions compared to controls (Fig. 2c; Table 2a, mANOVA F_(1,30)_ = 37.8855, *p* = 0.0000, n = 6 each). Further subcompartment comparisons revealed a similar Homer1a expression outcome in the HBO cohort as had been detected in the wLFS + HBO cohort: HBO significantly triggered an increase in Homer1a mRNA expression in the distal CA1 and proximal CA3 of the dorsal hippocampus (Fig. 2d; Table 2b, c). No significant changes in Homer1a expression were detected in the DG under these conditions. Our results confirm that acquisition of novel item-place knowledge triggers subcompartment-specific Homer1a mRNA expression in the cornus ammonis.

**Table 2:** Summary of statistical analysis of nuclear Homer1a expression in the hippocampus in the absence of afferent stimulation

	HBO versus control
*(a)*	
**CA1**	***p* = 0.0008**
**CA3**	***p* = 0.0002**
**DG**	*p* = 0.9932
*(b)*	
**dCA1**	***p* = 0.0001**
**pCA1**	*p* = 0.9877
**dCA3**	*p* = 0.9817
**pCA3**	***p* = 0.0001**

	HBO
*(c)*	
**dCA1 versus pCA1**	***p* = 0.0013**
**dCA3 versus pCA3**	***p* = 0.0001**

Statistical outcome of: a) between-group comparisons for each hippocampal subregion using Tukey HSD post hoc test; b) between-group comparisons for each subcompartment using Tukey HSD post hoc test; c) between- subcompartment comparisons using Tukey HSD post hoc test

Significant effects (*p* < 0.05) are highlighted in bold font. All groups include data from n = 6 animals

*dCA1* distal CA1, *dCA3* distal CA3, *pCA1* proximal CA1, *pCA3* proximal CA3, *DG* dentate gyrus, *HBO* holeboard including objects

### Homer1a mRNA expression increases in an equivalent manner across the proximodistal axis of the cornus ammonis when LTD is induced solely by means of patterned afferent stimulation of the Schaffer collaterals. STD has no effect on Homer1a expression

To clarify to what extent the abovementioned Homer1a expression was triggered by wLFS (rather than by the novel HBO experience), we assessed nuclear IEG expression in animals that received wLFS in the absence of an item-place learning event. Overall, we found no significant changes in the relative expression of Homer1a mRNA in any hippocampal subregion (wLFS, Fig. 2a, e), including the distal and proximal regions of CA1 and CA3 (Fig. 2b), compared to controls (Table 1a, b). Comparisons with Homer1a expression in wLFS + HBO animals revealed that Homer1a expression in wLFS + HBO animals was significantly greater than in wLFS animals (Table 1a). These findings suggest that induction of STD at SC-CA1 synapses does not trigger information encoding in hippocampal subfields.

We then explored to what extent Homer1a expression is increased when LTD is induced solely by means of afferent stimulation (i.e. in the absence of HBO). We detected significant elevations of Homer1a expression in both the CA1 and CA3 subregions relative to control animals (LFS, Fig. 2a, e; Table 1a). A subsequent post-hoc test revealed a significant elevation of Homer1a mRNA expression in the hippocampal CA subfields, but not in the DG, following LFS (Table 1a, n = 6 each). This finding is consistent with the fact that Schaffer collateral stimulation occurs upstream of the DG. Further analysis across the CA subcompartments revealed that both the distal and proximal parts of the CA1 and CA3 regions exhibited a significantly greater expression of Homer1a mRNA compared to control animals (Fig. 2b, e; Table 1b). Interestingly, Homer1a mRNA expression in the distal and proximal parts of the CA subfields was similarly elevated (Fig. 2b, e; Table 1c), suggesting that LFS given at the SC-CA1 synapses recruits neurons in the CA1 and CA3 subfields to a comparable extent. Thus, the differentiation of information encoding by the distal CA1 and proximal CA3 subfields that occurs in the wLFS + HBO condition, is absent when LTD is induced solely by means of afferent stimulation.

### Hippocampal LTD that is facilitated by novel item-place learning triggers immediate early gene expression in the retrosplenial cortex

Given that the RSC supports spatial information processing (Stacho and Manahan-Vaughan 2022a; Chao et al 2022), we explored to what extent areas 29 and 30 of the anterior RSC are engaged when the hippocampus stores information in the form of LTD. For this we assessed nuclear Homer1a expression in the RSC in response to the abovementioned wLFS + HBO, and LFS protocols, both of which had induced LTD in SC-CA1 synapses.

**Fig. 3:**
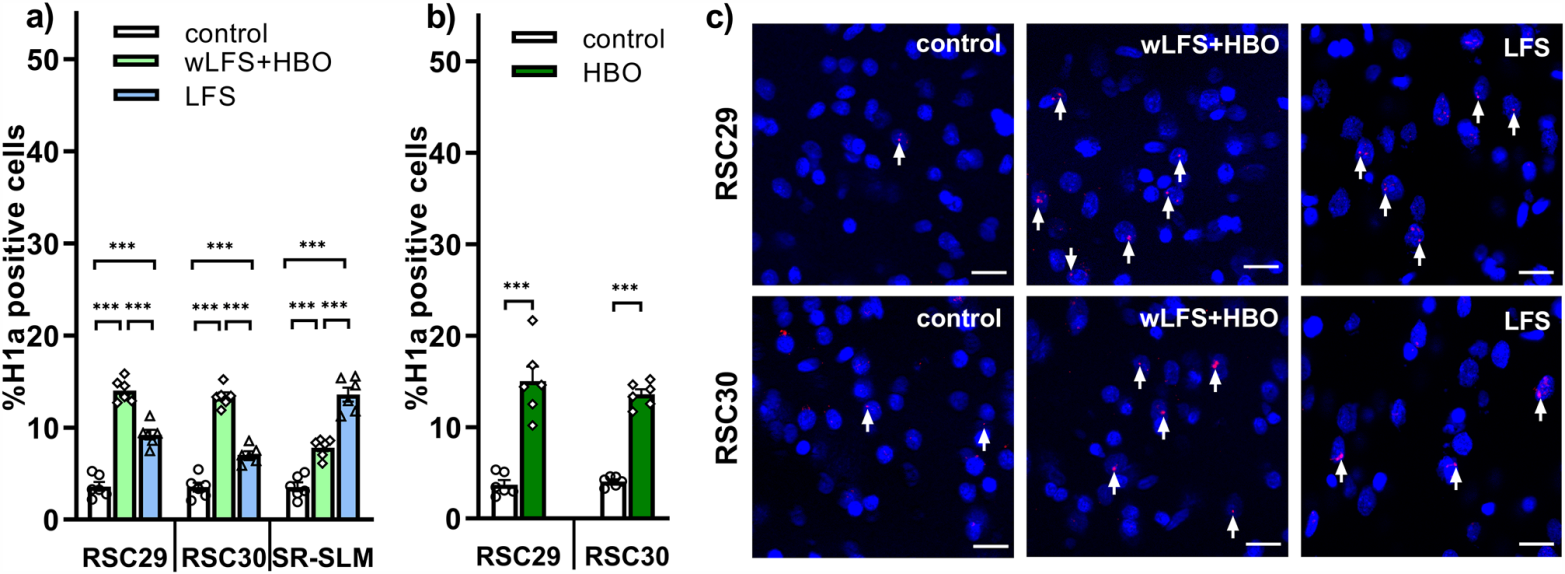
Nuclear Homer1a expression is increased in the retrosplenial cortex by induction of hippocampal LTD. **a** Bar charts show mean percentages of Homer1a mRNA positive nuclei in the anterior RSC areas 29 and 30 under different experimental conditions. Application of LFS at SC-CA1 synapses, that resulted in hippocampal LTD, significantly increased Homer1a mRNA expression in the RSC compared to controls. Exposure to a holeboard containing novel items inserted in the holeboard holes (HBO) during wLFS (wLFS + HBO), that also resulted in hippocampal LTD, resulted in a greater expression of Homer1a mRNA compared to animals that expressed LTD in the hippocampus as a result of LFS alone, as well as compared to controls. At the border of stratum radiatum (SR) and stratum lacunosum-moleculare (SLM), a significant increase in Homer1a mRNA expression was observed in LFS and wLFS + HBO animals, compared to control. ***p < 0.001. **b** In the absence of electrophysiological stimulation, novel acquisition of HBO significantly increased Homer1a mRNA in the anterior RSC areas 29 and 30 compared to controls. ***p < 0.001. **c** Photomicrographs show examples of increased Homer1a expression in the anterior RSC in response to wLFS + HBO and to LFS of SC-CA1 synapses, compared to responses in controls. Images were magnified to 20 ×. Nuclei were stained with DAPI (blue). Homer1a signals (red dots) are indicated by white arrows. Scale bar: 20 μm

In control animals we found that the nuclear expression of Homer1a mRNA was very low in RSC (Fig. 3a, c). Significant elevations of IEG expression occurred, however, when animals engaged in novel item-place learning during wLFS, or received LFS in the absence of learning (mANOVA of LFS versus wLFS + HBO versus control: F_(2,26)_ = 203.104, *p* = 0.000, n = 6 each).

The wLFS + HBO condition triggered significantly elevated Homer1a mRNA expression in RSC compared to control animals (Fig. 3a, c; Table 3a). No significant differences in relative Homer1a expression were detected between the RSC29 and RSC30, however (Table 3b), suggesting that this kind of information processing recruited equal proportions of neurons in the RSC29 and RSC30.

**Table 3:** Summary of statistical analysis of nuclear Homer1a expression in the retrosplenial cortex and at the border of stratum radiatum and stratum lacunosum-moleculare

	wLFS + HBO versus control	LFS versus control	wLFS + HBO versus LFS
*(a)*			
**RSC29**	***p* = 0.0001**	***p* = 0.0001**	***p* = 0.0001**
**RSC30**	***p* = 0.0001**	***p* = 0.0002**	***p* = 0.0001**
**SR-SLM**	***p* = 0.0004**	***p* = 0.0001**	***p* = 0.0001**

	wLFS + HBO	LFS
*(b)*		
**RSC29 versus RSC30**	*p* = 0.9187	***p* = 0.0290**

	HBO versus control (no electrophysiological stimulation)
*(c)*	
**RSC29**	***p* = 0.0001**
**RSC30**	***p* = 0.0001**

d)	
	HBO
**RSC29 versus RSC30**	*p* = 0.6755

Statistical outcome of between-group comparisons using Tukey HSD post hoc test (a, c), or between-subregion comparisons using Tukey LSD post hoc test (b, d)

Significant effects (*p* < 0.05) are highlighted in bold font. All groups include data from n = 6 animals

*RSC* retrosplenial cortex, *SR-SLM* stratum radiatum-stratum lacunosum-moleculare, *HBO* holeboard including objects, *LFS* low frequency stimulation, *wLFS* weak low frequency stimulation

In animals that received LFS to induce hippocampal LTD (> 4 h), we also detected significant increases in the expression of Homer1a mRNA in both RSC29 and RSC30 (Fig. 3a, c; Table 3a). Strikingly, Homer1a expression levels were significantly higher in the RSC29 than in the RSC30 (Table 3b). But, when we compared neuronal responses in the RSC under the two experimental conditions (LFS vs. wLFS + HBO), the proportion of activated neurons in the RSC following LFS was significantly lower than IEG expression triggered by wLFS + HBO (Table 3a). These results indicate that the RSC engages in information encoding when LTD is induced in the hippocampus by means of LFS of the Schaffer collaterals. However, the involvement of the RSC in the encoding of hippocampus-generated information is stronger when hippocampal LTD is facilitated by spatial learning.

Given that the main input from CA1 to the RSC29 originates from neurons located at the border of the stratum radiatum and stratum lacunosum-moleculare (Miyashita and Rockland 2007), we examined neuronal activation in this cell population (SR-SLM, Fig. 3a). We detected a significant elevation of Homer1a mRNA expression in the LFS and wLFS + HBO cohorts, compared to controls. Moreover, the neuronal response triggered by LFS alone was significantly greater than response triggered by wLFS + HBO (Fig. 3a; Table 3a). These data indicate that interneurons located in SR-SLM area are recruited during the induction of LTD in CA1.

Additionally, in the control cohort (without electrode implantation), novel exposure to HBO significantly increased Homer1a mRNA expression in both RSC29 and RSC 30 compared to controls (that underwent no exploration event) (Fig. 3b; Table 3c). No significant differences in subregional expression of Homer1a were observed in the RSC (Table 3d). We did not detect any significant difference between the proportion of neurons in the RSC activated by novel acquisition of HBO (in the absence of electrophysiological stimulation) and those activated by the combination of wLFS + HBO (one-way ANOVA, F_(1,20)_ = 0.4502, *p* = 0.5099). Significantly greater expression of Homer1a in both RSC29 and RSC30 occurred following HBO in the absence of wLFS compared to RSC IEG expression after induction of LTD in CA1 by means of LFS (one-way ANOVA, F_(1,20)_ = 46.5607, *p* = 0.0000). These results indicate that in both areas 29 and 30 of the anterior RSC, novel acquisition of HBO triggers nuclear IEG encoding that is more robust than encoding induced by LFS.

## Discussion

In this study, we report that LTD that is facilitated by weak afferent stimulation of the Schaffer collaterals during item-place learning, specifically triggers nuclear immediate early gene expression (IEG) in the distal-CA1 and proximal-CA3 regions of the dorsal hippocampus, as well as in areas 29 and 30 of the retrosplenial cortex (RSC). The dentate gyrus is completely unaffected by this kind of information processing and storage. Strikingly, the discrete effects on nuclear IEG expression in subfields of the proximodistal axis of the cornus ammonis are completely obliterated when LTD is induced by sole stimulation of the Schaffer collaterals (SC). In this case, IEG expression occurs across the entirety of the cornus ammonis. Interestingly, the RSC exhibited lower nuclear IEG expression following electrophysiological LTD induction, compared to LTD that was facilitated by item-place learning, although in this case a higher level of IEG expression was detected in RSC29 compared to RSC30.

The two-stream stream hypothesis (Mishkin et al. 1983) proposes that visual ‘what’ and ‘where’ information is processed separately by cortical structures. According to this hypothesis, the ventral stream, that originates in the visual cortex and runs along the ventral surface to the temporal cortex, subserves the encoding and processing of identity of objects (“what” information), whereas the dorsal stream, that extends along the dorsal axis into the parietal cortex, processes information related to the spatial location of the objects (“where” information) (Mishkin et al. 1983). By means of this segregation of ‘what’ and ‘where’ information, spatial and non-spatial information can be discriminated from each other (Hafting et al. 2005; McNaughton et al. 2006; Sargolini et al. 2006; Moser and Moser 2008; Solstad et al. 2008). Interestingly, RSC, with its well-positioned and reciprocal connections to different important brain structures, is involved in both spatial and non-spatial information processing (Vedder 2017; Fischer et al. 2020; Landeta et al. 2020; Chao et al. 2022).

Although the dorsal stream hypothesis was initially proposed to address aspects of visual information processing at the cortical level (Mishkin et al. 1983), later studies indicated that the hippocampus is affected by information transmission along the dorsal and ventral streams (Hampson et al. 1999; Knierim et al. 2006; Ito and Schuman 2012), and that ‘what’ and ‘where’ information flow, via entorhinal cortical projections to the hippocampus, targets very specific subfields of the proximodistal axis of the cornus ammonis (Naber et al. 2001; McNaughton et al. 2006; Deshmukh and Knierim 2011; Ito and Schuman 2012; Nakamura et al. 2013). In fact, even the dentate gyrus (DG) engages to some degree in the discrimination of ‘what’ and ‘where’ information (Chawla et al. 2005; Hoang et al. 2018, 2021). From these findings, it has become clear that spatial information inevitably contains both ‘what’ and ‘where’ information, and that this information is integrated into spatial representations by the hippocampus (Stacho and Manahan-Vaughan 2022b). Spatial content learning appears to predominantly drive the encoding of ‘what’ aspects of spatial experience by the hippocampus. For example, novel exposure of rats to constellations of large landmark items in an environment drives nuclear IEG expression in the proximal CA3 region and in the DG, whereas novel exploration of less overt items (that must be physically approached in order to be found, such as in the HBO paradigm used in the present study) drives nuclear IEG expression in the proximal CA3 and the distal CA1 regions. These subfields have been ascribed a role in the processing of ‘what’ information (Tamamaki and Nojyo 1995; Naber et al. 2001; Deshmukh and Knierim 2011; Ito and Schuman 2012; Nakamura et al. 2013). The abovementioned differentiation of IEG expression in the cornus ammonis or DG, based on item dimensions, their spatial characteristics, and their overt manifestations, also indicates that different subfields of the hippocampus differentiate salient metric aspects of items in space.

These findings, derived from experience-dependent gene-trap experiments, align exactly with findings with regard to the promotion of expression of synaptic plasticity by spatial content learning: whereas novel learning about spatial constellation of landmarks, enables LTD in the DG and mossy fiber-CA3 synapses, novel learning about spatial constellations of discrete, less overt, items facilitates LTD in commissural associational (AC)-CA3 synapses and SC-CA1 synapses (Stacho and Manahan-Vaughan 2022b). This suggests not only, that the ‘what’ element of spatial representations is tightly associated with the expression of LTD, but also that discrete hippocampal subfields engage in encoding of these facets of spatial content. Our current study furthers our knowledge in this regard. We found that LTD that is facilitated by discrete item-place exploration, drives nuclear IEG expression in the proximal CA3 and the distal CA1 regions, without affecting the dentate gyrus. The proximal and distal segments of hippocampal cornus ammonis (CA) are believed to process item information in a differentiated manner, mostly mediated through the entorhinal area. The proximal CA1 region receives input via the distal CA3 regions that conveys spatial information (Hafting et al. 2005; McNaughton et al. 2006; Sargolini et al. 2006; Moser and Moser 2008; Solstad et al. 2008). By contrast, neurons in the distal CA1 region receive inputs from the proximal CA3 that deliver predominantly non-spatial information (Tamamaki and Nojyo 1995; Naber et al. 2001; Deshmukh and Knierim 2011; Ito and Schuman 2012; Nakamura et al. 2013). Although inputs to the CA3 region from the medial and lateral entorhinal cortices are not topographically organized (Nilssen et al. 2019), spatial information processing differs along the proximodistal axis of CA3, as reflected by the occurrence of sharper place field representations in proximal compared to distal CA3 (Lu et al. 2015). This may support the differentiation of information processing along the proximodistal axes of CA3 and CA1, whereby the proximal CA3 and distal CA1 subcompartments of the cornus ammonis are proposed to process information from the ‘what’ pathway (item features) and the distal CA3 and proximal CA1 process ‘where’ information (Nakamura et al. 2013; Sauvage et al. 2013; Flasbeck et al. 2018). In the present study, Homer1a mRNA expression was significantly increased in precisely these ‘what’ subcompartments (e.g. proximal CA3 and distal CA1) when animals explored small item-place configurations in the presence, or absence, of electrophysiological stimulation. This suggests that wLFS was not causative of this response, rather the learning event facilitated LTD. The IEG expression distribution that we detected is in line with previous reports that the proximal CA3—distal CA1 network processes ‘what’ information (Ito and Schuman 2012; Nakamura et al. 2013; Hoang et al. 2018). The fact the nuclear IEG expression was increased, signifying information encoding, aligns with the findings of previous studies that showed that this kind of novel item-place exposure triggers learning in the cornus ammonis (Manahan-Vaughan and Braunewell 1999; Kemp and Manahan-Vaughan 2004; Popkirov and Manahan-Vaughan 2011; Hagena and Manahan-Vaughan 2011).

It was striking that the electrophysiological induction of LTD by means of prolonged low frequency stimulation resulted in nuclear IEG expression across all areas of the cornus ammonis. This general and indiscriminate pattern of expression in the hippocampus bears no resemblance to the localised pattern of expression triggered by the coupling of weak afferent stimulation with novel item-place experience.This finding is similar to a previous study that showed that induction of either LTD, or LTP, at perforant path-dentate gyrus synapses drives nuclear gene encoding in a non-differentiated manner, across the entire dorsal hippocampus (Hoang et al 2021). Both studies cast into question to what extent synaptic plasticity that is induced in the absence of a learning event can reveal the precise role of, and cellular mechanisms underlying, long-term information encoding by means of synaptic plasticity. A comparison of both studies reveals another interesting aspect: Induction of either LTP, or LTD, at DG synapses by spatial learning events results in downstream information encoding in the CA3 and CA1 regions (Hoang et al 2021), suggesting that learning-based synaptic information encoding by the DG has a knock-on effect on information encoding in the cornus ammonis. By default, this means that information that is encoded by the DG will be integrated to some extent into representations generated by the CA3 and CA1. By contrast, our current study revealed that LTD that is enabled by prolonged afferent stimulation of the SC, or by item-place learning during mild SC stimulation, has no effect whatsoever on IEG expression in the DG. This suggests that the DG is not ‘kept in the loop’ about information processing in the CA1 region. In addition, our findings support a recent hypothesis that proposes that LTP is induced rapidly upon exposure to novel space or a generalised change in space, whereby landmark information is integrated in DG and DG-CA3 synapses by means of subfield specific LTD, and detailed more discrete, item information is integrated in associational/commissural fiber-CA3 and SC-CA1 synapses by means of LTD (Stacho and Manahan-Vaughan 2022b). These processes emerge temporally as the animal spends time in the environment and can progressively register landmark features, or physically approach more subtle features of space. By this means the initial LTP representation is rendered more unique, more robust and more resistant to generalization (Stacho and Manahan-Vaughan 2022b).

Our findings align with the synaptic tagging and capture hypothesis (Frey and Morris 1997). According to this hypothesis, afferent stimulation protocols, used to induce persistent forms of synaptic plasticity in the hippocampus, lead to the setting of a local tag and the synthesis of plasticity related proteins (PRPs), which are then captured by the local tag to restructure the affected synapse. These factors are required for the maintenance of long-term potentiation (Redondo and Morris 2011). By contrast, weak afferent stimulation, that fails to result in persistent plasticity, triggers the setting of a synaptic tag, but not PRP synthesis (Redondo and Morris 2011). In the absence of PRPs, the synaptic tags fade, and synaptic strength drops back to naïve levels (Redondo and Morris 2011). Although this hypothesis mainly addresses mechanisms underlying persistent forms of LTP, synaptic tagging has also been reported for hippocampal LTD (Sajikumar et al. 2007). In the current study, we showed that LFS of Schaffer collaterals leads to LTD that lasts for > 24 h, whereas weak LFS only triggers transient synaptic depression which lasts < 90 min. LFS (that induces LTD), but not weak LFS (that induces STD), elevated nuclear Homer mRNA expression in the hippocampus, suggesting that only in the former case PRPs were synthesized. These data are also consistent with other findings that long-term plasticity, but not short-term plasticity, requires ongoing macromolecular synthesis (Schwartz et al. 1971; Castellucci et al. 1980; Tang et al. 2002; Abraham and Williams 2008).

The impact of induction of hippocampal LTD (in freely behaving animals) on information processing in the RSC is, as yet, unclear. In the present study, we observed that LFS at the Schaffer collaterals that triggered LTD in the CA1, significantly enhanced Homer1a mRNA expression in both RSC29 and 30. This finding is in line with anatomical evidence that a functional connectivity exists between the hippocampal formation and the RSC (Vogt and Miller 1983; Finch et al. 1984; Naber and Witter 1998; van Groen and Wyss 2003; Miyashita and Rockland 2007). Interestingly, and unlike the result obtained in the hippocampus, where equal proportions of neurons in all subcompartments of the CA regions were activated by this stimulation protocol, neurons in the RSC29 expressed more IEG compared to neurons in the RSC30, when LFS was applied to induce hippocampal LTD. This IEG response agrees with anatomical studies reporting that the subiculum and CA1 projections terminate in RSC29, whereas only the subiculum sends projections to RSC30.

According to anatomical data, the hippocampal projections to RSC29 originate in neurons of the stratum pyramidale and from the border between stratum lacunosum-moleculare and stratum radiatum (Meibach and Siegel 1977; van Groen and Wyss 1990b, 2003; Naber and Witter 1998; Miyashita and Rockland 2007): the site of induction of LTD in the present study was the stratum radiatum. Strikingly, compared to responses triggered by item place learning in conjunction with weak LFS, prolonged LFS triggered an increase in neuronal IEG expression in interneurons that are located at the border of stratum radiatum and stratum lacunosum-moleculare. Given the reported role of interneuronal GABA in hippocampal LTD induction (Thiels et al. 1994) this raises the question as to whether potent LTD induction can lead to modifications of interneuronal circuitry (Nishiyama et al. 2010), or the output of the CA1 region to the RSC (McMahon and Kauer 1997; Yanovsky et al. 1997). Our observation, together with these anatomical aspects, could explain why Homer1a expression was greater in RSC29 compared to RSC30 when LTD was induced in the stratum radiatum of CA1.

Little is known about how the RSC responds to ‘what’ or ‘where’ learning events at the level of nuclear IEG expression. In the present study, when LTD was enabled via item-place learning, significantly greater expression levels of Homer1a mRNA were evident in the nuclei of neurons of the entire RSC, compared to responses generated by induction of LTD solely by means of afferent stimulation. This suggests that item-place learning triggered a different and more robust kind of encoding in the anterior RSC, than that triggered by hippocampal LTD. Moreover, when we compared the effects of item-place learning alone with the effects of wLFS in conjunction with item-place learning, the elevations of nuclear Homer1a expression were equivalent in the RSC under both conditions. This suggests that weak LFS in the hippocampus is not needed for the information encoding triggered in the RSC by novel item-place learning. Given the very robust IEG expression triggered by item-place learning in this structure, this raises the suggestion as to whether the hippocampus informs the RSC about this spatial learning experience or vice versa.

Item-place information storage inevitably includes information about item identity (‘what’) and item location (‘where’) (Chao et al. 2022). The accurate processing of this kind of information necessitates the integration of idiothetic information (head position, path direction) and allocentric information (spatial orientation and spatial referencing relative to external environmental features). Thus, the non-specific increases in Homer1a expression triggered in the RSC by item-place learning may align with roles ascribed to the RSC in related forms of information processing: whereas RSC30 is largely involved in processing allocentric (‘where’) information, RSC29 is believed to process both idiothetic and allocentric information (Vann and Aggleton 2005; Hindley et al. 2014b, 2014a; Landeta et al. 2020). This of course, raises an interesting question as to the apparent differences in the sites of information encoding triggered by learning-facilitation of LTD and by item-place learning (without wLFS), as detected in the present study. The localised expression of Homer1a in distal-CA1 and proximal-CA3 point towards a prioritization of the encoding of ‘what’ elements of the item-place experience by the hippocampus (Naber et al. 2001; Ito and Schuman 2012; Nakamura et al. 2013). By contrast, based on the abovementioned role of RSC 29 and 30 in allocentric and idiothetic information processing, the RSC engaged in encoding of both ‘what’ and ‘where’ components of this experience. It is, however, difficult to extricate ‘what’ from ‘where’ information in an item-place event. In another study it was shown that it is, in fact, the spatial configurations of the items that facilitated CA1 LTD (Kemp and Manahan-Vaughan 2004). But the results of the present study suggest that the RSC is not simply duplicating the role of the hippocampus in this experience-dependent information encoding event. Another interesting finding of this study is that the RSC not only processes this kind of information, but also *encodes* information that is actively stored at hippocampal synapses. This observation may reflect the role of RSC as an interface structure between the cortical areas and the hippocampal formation that support learning and memory (Reep et al. 1994; Warburton et al. 2001; Vann et al. 2009; Olsen et al. 2017). Moreover, evidence exists that the RSC is able to store and retrieve spatial memory (Miller et al. 2014; Vedder 2017; Milczarek et al. 2018).

To what extent the effects we detected are specific to Homer1a remains to be explored. Homer1a plays an important role as an activity-dependent physical interface between the postsynaptic density and PRPs such as the N-methyl-D-aspartate receptor (NMDAR) (Moutin et al. 2012). It also serves as an intermediary between group I metabotropic glutamate receptors and NMDARs, that in turn can promote synaptic plasticity (Bertaso et al. 2010; Sylantyev et al. 2013). Transcription of Homer1a is triggered throughout the brain by LTP (Kato et al. 1997, 1998) and novel spatial learning (Vazdarjanova et al. 2002; Marrone et al. 2008; Clifton et al. 2017; Hoang et al. 2018, 2021). Nonetheless, it is not the only IEG that responds to item-place learning, induction of hippocampal plasticity, or learning facilitated plasticity: nuclear Arc expression is triggered by landmark configuration learning in the DG (Hoang et al. 2018) and by learning-facilitated plasticity in the DG (Hoang et al. 2021). Learning-facilitated LTD at SC-CA1 synapses also requires cFos (Kemp et al. 2013).

## Conclusions

The results of this study highlight the contribution of the cornus ammonis and the RSC to the processing of hippocampal LTD that is enabled by hippocampus-dependent learning. In particular, the distal CA1 and proximal CA3 subfields expressed significant elevations of nuclear Homer1a expression when LTD was facilitated by de novo item-place learning. This suggests in turn, that ‘what’ information is a prominent facet of item-place learning. Moreover, significant Homer1a expression was detected in both RSC29 and 30 as a result of the induction of LTD at SC-CA1 synapses, and by item-place learning. These data indicate that the RSC is part of an information storage network that is engaged when experience-dependent information encoding is triggered by spatial content learning.

## Data Availability

The data that support the findings of this study are available from the corresponding author upon reasonable request.
